# Sex and the streets: the open secret of sexual abuse among Pakistan’s two million street children

**DOI:** 10.1186/s13034-021-00420-3

**Published:** 2021-11-13

**Authors:** Amir Humza Sohail, Muhammad Hassaan Arif Maan, Sachal Sohail

**Affiliations:** 1 Department of Surgery, New York University Winthrop Hospital, New York, USA; 2 Medical College, The Aga Khan University, Stadium Road, Karachi, Pakistan; 3 King Edward Medical University, Lahore, Pakistan

**Keywords:** Street children, Sexual abuse, Human immunodeficiency virus, Sexual exploitation

## Abstract

**Background:**

About two million children live on the streets in Pakistan. Their complicated past and dire living conditions make them susceptible to many psychological and physical problems, including sexual abuse.

**Main body:**

With little research on the topic, the prevalence of sexual intercourse among street children is reported to be as high as 88% in Pakistan. With commercial sex a common practice among the street children, public places such as bus terminals and parks have become foci of prostitution and sexual exploitation. A growing concern is the spread of HIV/AIDS among the affected children due to a general lack of awareness about the disease and its prevention and high prevalence of unsafe sexual practices. The generally apathetic attitude of the society towards this issue and the affected children, combined with a lack of commitment and limited resource allocation by the government, has contributed in deteriorating the situation further.

**Conclusions:**

A comprehensive multi-pronged strategy involving government, societal and international stakeholders is crucial to tackle the current crisis.

## Background

It is estimated that around two million children live on the streets in Pakistan. Usually, these children experience complex dire circumstances during both pre- and post-homelessness periods [1]. This makes them highly vulnerable to an array of psychological and physical issues, particularly sexual abuse.

The prevalence of sexual abuse among street children is distressingly high worldwide. Importantly, the patterns of survival sex, including multiple partners and unprotected intercourse, in Pakistan are strikingly similar to those seen globally, with studies from Africa, Eastern Europe and South Asia showing similar findings [2, 3]. Unfortunately, until recently, despite the scale of the crisis, it has received minimal government attention and interventions to alleviate the situation are lacking. Further, major gaps exist in research on effective strategies to prevent sexual exploitation of street children, provide for their psychological needs, and ensure long term rehabilitation.

While there is paucity of high-quality evidence on the topic in Pakistan, the prevalence of sexual intercourse among street children in Pakistan has been reported to be as high as 88%, with up to 66% of those sexually active reporting sex with adults too [2, 3]. One study reported 83% of the children to be sexually active within the last month, with a median age of 11 years at initiation of sexual activity (age at initiation in street children globally ranges from 10 to 16 years) [4, 5]. Alarmingly, 76% of children had been raped, mostly multiple times, while the same percentage, 76%, reported raping other children [4].

## Main text

Commercial sex among Pakistan’s street children is very common (53% reported selling sex in one study). Surprisingly, prostitution and sexual exploitation of street children is common in public places, such as bus terminals and parks. Evidence from India shows that factors such as lack of contact with family, being an orphan, night stay at public places are associated with sexual abuse among street children [6]. In Pakistan, children from nomadic families commonly resort to part-time commercial sex, mainly as a result of family pressure to earn more cash, while mockery from peers in case of boys subjected to sodomy once, and living on the streets for > 4 years are other factors associated with sexual activity [7, 8].

A major area of concern pertaining to sexual abuse among street children is HIV/AIDS [9]. HIV prevalence among street children, as evidenced in Russia, Iran, and South America, is several times higher than in the general population [6, 10–13]. In Pakistan, a general lack of awareness about the disease and its prevention, as well as unsafe sexual practices are common [4]. In one study, only 35% of participants had heard of HIV/AIDS, only 25.6% identified sexual intercourse as a method of transmission, and the majority (71%) were unaware of any prevention methods, which points towards absence of education programs for these children. Further, multiple sexual partners (mean 4.2), low prevalence (20%) of personal realization of being at-risk, high prevalence (72%) of sexual activity with male partners, including receptive intercourse (69%), and a host of other factors put these children at risk of contracting HIV [4]. Alarmingly, barrier protection use in commercial sexual transactions is almost non-existent; only 10% used condoms with male partners [4].

Despite the magnitude of the crisis, efforts to tackle the issues surrounding sexual abuse among Pakistan’s street children are few and far between. A lack of resolve and political will by the government to intervene, weak criminal justice system and limited resources dedicated towards rehabilitation of street children in general are major challenges [14]. This is further complicated by Pakistan’s complex and volatile socio-political milieu, and underdeveloped child protection systems, which make high quality qualitative and quantitative data gathering on this issue a challenging, “complex and technical exercise” [15]. Moreover, major research gaps on gender relations and sexuality in early years in the Pakistani context exist, which hinder a detailed understanding of power dynamics and sexual activity among street children [7].

In recent years, there has been some progress. For example, in March 2020, after a series of high-profile cases, the government passed legislation on child abuse, which besides establishing life sentence for child abuse offences also provides a framework for establishment of a national database on missing children and stipulates roles of various law-enforcement agencies in the legal process; prior to this there was no legislation pertaining to child abuse [16]. However, the absence of will and attention to detail is obvious from the fact that despite repeated promises basic, yet crucial, measures, such as creation of a much-publicised publicly available sex offender registry, have not been completed yet.

To improve the dire conditions, concerted efforts in multiple other areas are crucial. From a societal standpoint, a collective sense of apathy towards street children where they are often viewed as social outcastes—especially certain subgroups such as nomads, and a poor social understanding of sexual abuse limited to the concept of rape and sodomy are other factors that impede progress on the issue of sexual abuse among street children [7, 9, 17]. Sociocultural and religious reasons leading to portrayal of sex as a taboo subject, and a culture of silence and ‘shame and family dishonor’ about sexual abuse not only lead to underreporting but also hinder public discourse and thus societal realization of the gravity of sexual abuse among children in Pakistan [6, 17].

Poverty and economic deprivation, often resulting from death of parents or conflict with extended family, along with other factors leads to children living on the streets which in turn leads to survival sex [17]. Thus, to tackle the problem of sexual abuse among street children, steps should focus on elimination of factors leading to such a large population of street children, as well as reunion with family and rehabilitation [18]. Open communication between parents/caretaker and children regarding sexual abuse with the aim to increase children’s knowledge regarding abuse creates an empowering environment with higher disclosure rates and boosts children’s self-esteem, and has been shown to be a significant deterrent to perpetrators [19, 20]. However, given societal values where open discussion about sex is frowned upon, this is not the norm in Pakistan. Similarly, there is a need to include sexual abuse in personal protection education in formal school curricula [7].

We recommend a focus on research investigating development of gender power dynamics, masculinities and sexuality in Pakistani adolescents and children that will provide powerful insights to develop appropriate mechanisms and structures to cater to the needs of street children, as well as robust data gathering, such as on child sex offender. Community education initiatives using a range of media to reach all strata of society are of particular importance, and can facilitate identification of at-risk children and offenders, reunification of street children with families, societal reintegration and greater acceptance of street children, and change the culture of silence, shame and stigma surrounding sexual abuse [7]. Initiation of awareness and life skills training programs for at-risk street children can help them identify and avoid high-risk situations, increase safe sex practices and lead to higher rates of reporting. We believe that these recommendations may also be applicable to other societies with similar sociocultural norms, such as India, where the problem of sexual abuse among street children is prevalent [6, 14].

## Conclusions

The issue of sexual abuse among Pakistan’s two million street children has been ignored by policymakers for too long. It is high time that a comprehensive multi-pronged strategy involving government, societal and international stakeholders be devised to tackle the current crisis. This strategy must be informed by high-quality local evidence on factors contributing to and exacerbating the situation as well as interventions aimed at rehabilitating Pakistan’s street children.

Awareness campaigns to highlight the severity and extent of child sex abuse, particularly among street children, and its impact on the affectees’ physical and mental health, investment in institutions to train mental health professionals able to cater to their psychological needs, as well as education of at-risk children are paramount. Finally, the importance of strict implementation of the recent legislation on child sexual abuse to ensure deterrence, identification and monitoring of previous offenders, and evaluation and continuous improvement of newly established legal framework to recover missing children and rehabilitate them cannot be overestimated.

## Data Availability

Not applicable.

## Abbreviations

HIV: Human Immunodeficiency Virus
AIDS: Acquired Immune Deficiency Syndrome
